# Immunomodulatory Activity of Carboxymethyl Pachymaran on Immunosuppressed Mice Induced by Cyclophosphamide

**DOI:** 10.3390/molecules26195733

**Published:** 2021-09-22

**Authors:** Feng Liu, Lijia Zhang, Xi Feng, Salam A. Ibrahim, Wen Huang, Ying Liu

**Affiliations:** 1 College of Food Science and Technology, Huazhong Agricultural University, Wuhan, 430070, China; liufeng163fl@163.com (F.L.); 13297021290@163.com (L.Z.); huangwen@mail.hzau.edu.cn (W.H.); 2 Department of Nutrition, Food Science and Packaging, San Jose State University, San Jose, CA 95192, USA; xi.feng@sjsu.edu; 3 Department of Family and Consumer Sciences, North Carolina A&T State University, 171 Carver Hall, Greensboro, NC 27411, USA; ibrah001@ncat.edu

**Keywords:** *Poria cocos*, carboxymethyl pachymaran, immunomodulatory, cyclophosphamide, gut microbiota

## Abstract

The effects of immunomodulatory activity of two types of carboxymethyl pachymaran (CMP-1 and CMP-2) on cyclophosphamide (CTX)-induced mice were investigated. Both CMP-1 and CMP-2 were found to restore the splenomegaly and alleviate the spleen lesions and the mRNA expressions of TLR4, MyD88, p65 and NF-κB in spleen were also increased. CMP-1 and CMP-2 could enhance the immunity by increasing the levels of TNF-α, IL-2, IL-6, IFN-γ, Ig-A and Ig-G in serum. In addition, CMP-1 could increase the relative abundance of *Bacteroidetes* and reduce the relative richness of *Firmicutes* at the phylum level. CMP-1 and CMP-2 could reduce the relative abundance *Erysipelatoclostridum* at the genus level. CMP-1 and CMP-2 might enhance the immune function of immunosuppression mice by regulating the gene expression in the TLR4/NF-κB signaling pathway and changing the composition and abundance of the intestinal microbiota. The results suggested that CMP-1 and CMP-2 would be as potential immunomodulatory agents in functional foods.

## 1. Introduction

The immune system is a rigorous defense mechanism against pathogens, damages, infections and pollutants [1], and is mainly composed of immune organs, immune cells and immune molecules. Disorder of the immune system may lead to excessive inflammatory reaction, autoimmune diseases and even cancer [2,3]. Cyclophosphamide (CTX) is an alkylating agent, which is used as a chemotherapeutic agent in various tumors, cancers and bone marrow transplantation to alleviate bacterial translocation and infection by mucosal barrier damage as well as other complications caused by chemotherapy combined with antibiotics [4]. However, there is evidence that CTX can increase myelosuppression and immunosuppression, interfering with the development and maturation of the immune system [5,6,7]. Therefore, the model of immunosuppressive mice is often established by CTX to evaluate the immunoregulatory activity of active ingredients.

*Poria cocos* is a common medicinal and edible resource, which is widely used in China and other Asian countries to alleviate joint pain, and improve the functionality of spleen, stomach and kidney, etc. The bioactive components in *Poria cocos* include polysaccharides, triterpenes, fatty acids, etc. The main bioactive component in *Poria cocos* is polysaccharide, based on biochemical and pharmacological studies, and has a wide range of biological activities including antitumor, immunomodulation, anti-inflammation, anti-oxidation, anti-aging, anti-hepatitis, anti-diabetes and anti-hemorrhagic fever effects [8,9,10]. However, *Poria cocos* are usually discarded as drug residue because of its poor solubility [11]. Molecular modification and structural improvement can change the chemical structures and physicochemical properties of polysaccharides (such as solubility, branching degree, molecular weight and structure conformation) [12], which are important parameters to explain the physiological activities of polysaccharides. The modification methods of polysaccharides are acetylation, sulfation, benzyolation, phosphorylation, selenylation, carboxymethylation, hydroxypropylation, partial hydrolysis and C-glycosylation, and some of these modification methods can improve the immunomodulatory properties of polysaccharides [13]. It has been reported that sulfated Chinese yam polysaccharides (S-CYP) increased the thymus index and proliferation of splenic lymphocytes of the mice compared with the cyclophosphamide (Cy)-induced immunosuppressed mice, and could restore the splenomegaly and promote the levels of splenic lymphocytes cytokines and immunoglobulin (Ig-G and Ig-M) secretion in serum. These results showed that S-CYP can serve as a promising immunomodulator [14]. Phosphorylation of *Radix Cyathulae officinalis* Polysaccharides increased serum immunoglobulin (Ig-G, Ig-A, Ig-M) concentrations, enhanced splenocyte proliferation and the thymus and spleen indices, promoted phagocytosis in peritoneal macrophages and enhanced cytokine serum levels, and increased the proportions of selected T cell subpopulations, indicating that phosphorylation of the polysaccharides promoted their immune-enhancing effects [15]. Carboxymethylation is one of the most common molecular modification methods, which is the replacement of a hydroxyl group of the polysaccharide by a carbonyl methyl group [16,17,18]. Wang, Yang, Gao, Wang, and Cao [19] prepared Carboxymethyl pachymaran (CMP) from *Poria cocos* through carboxymethylation, which had better water solubility and physiological activities.

Our previous study showed that two types of polysaccharides (CMP-1 and CMP-2) isolated from carboxymethyl pachymaran had immunomodulatory activity on RAW264.7 in vitro [20]. However, the in vivo immunomodulatory activity of CMP-1 and CMP-2 has not been studied yet. The aims of this study were to investigate the immunomodulatory activities of CMP-1 and CMP-2 on CTX-induced mice and to further understand the immunomodulatory mechanisms, including the pathway of gene expression and the changing of gut microbiota.

## 2. Results

### 2.1. Effects of CMPs on Immune Organ Index

**Figure 1:**
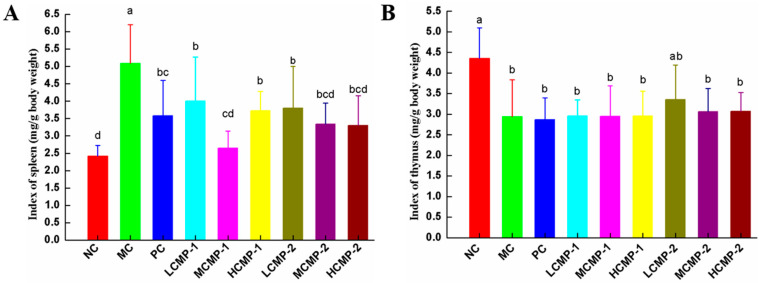
Effects of CMP-1 and CMP-2 on spleen index (**A**) and thymus index (**B**). The data were expressed as means ± SD (n = 10). Different superscript small letters (a–d) are significantly different (*p* < 0.05).

As shown in Figure 1, the spleen indexes for the MC group significantly increased (*p* < 0.05), and the thymus indexes significantly decreased (*p* < 0.05) when compared with those of the NC group. Compared with the MC group, the administration of lentinan, CMP-1 and CMP-2 significantly reduced the spleen indexes (Figure 1A). However, both lentinan and CMP had no effect on the thymus indexes (Figure 1B). With different doses of CMP administration, the spleen index was remarkably (*p* < 0.05) reduced and the middle dose of CMP-1 (100 mg/kg·bw/d) restored the spleen index to a normal level. The results indicated that CMP-1 and CMP-2 could promote the development of spleen tissues to relieve the degree of splenomegaly induced by CTX.

### 2.2. Effects of CMPs on Spleen Tissue Morphology of Mice

**Figure 2:**
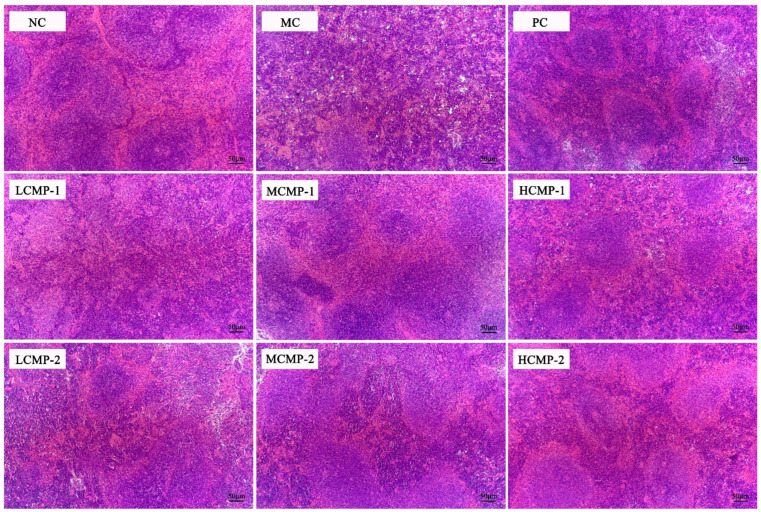
Effects of CMP-1 and CMP-2 on the morphology of the spleens of mice (HE, 200×).

The HE images of spleen were shown in Figure 2. The spleen cells of the NC group were tight and arranged well with a clear nucleus, and the boundaries of the red and white pulp of spleen were clear. After the CTX treatment, the spleen cell arrangement, and the red and white pulp of spleen in the MC groups exhibited unclear boundaries, specifically, the white pulp was destroyed and the trabeculae of spleen were dense but the number of lymphocytes was fewer. The red pulp and white pulp of the spleen in both CMP-1 and CMP-2 groups were clearer and their original morphology of spleen tissue were gradually recovered as well as the lymphocytes were increased when it was compared with MC group. The results indicated that CMP-1 and CMP-2 could alleviate the spleen lesions and repair the damage induced by CTX.

### 2.3. Effects of CMPs on Cytokine Production in Serum

**Figure 3:**
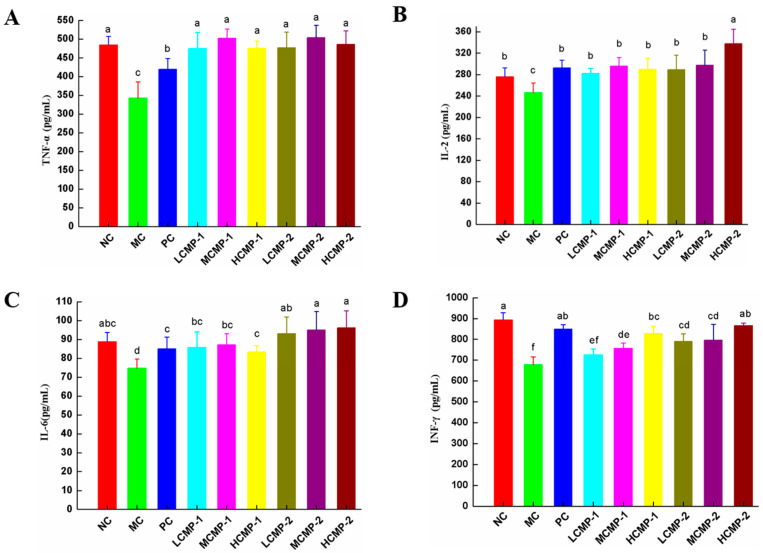
Effects of CMP-1 and CMP-2 on cytokine TNF-α (**A**), IL-2 (**B**), IL-6 (**C**), INF-γ (**D**) in serum. The data were expressed as means ± SD (n = 10). Different superscript small letters (a–f) are significantly different (*p* < 0.05).

As shown in Figure 3, the secretion of cytokines (TNF-α, IL-2, IL-4 and IFN-γ) were detected by ELISA kit to evaluate the immunoregulatory activities of CMP-1 and CMP-2. Compared with the NC group, the levels of TNF-α, IL-2, IL-6 and IFN-γ in the MC group were significantly decreased (*p* < 0.05), indicating that CTX can depress the immunocyte-mediated action. The administration of CMP-1 and CMP-2 significantly increased the levels of TNF-α, IL-2, IL-6 and IFN-γ. The results indicated that CMP could restore the secretion of cytokines in immunosuppressed mice.

### 2.4. Effects of CMPs on the Levels of Immunoglobulin in Serum

**Figure 4:**
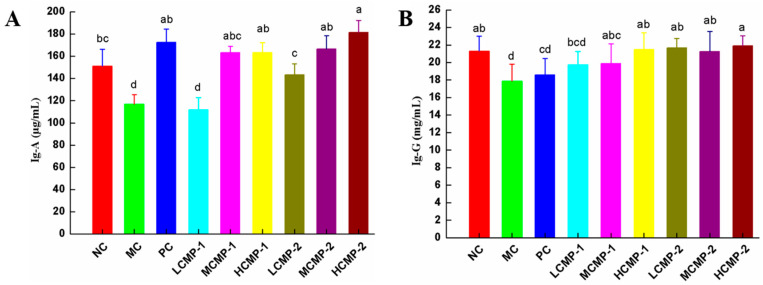
Effects of CMP-1 and CMP-2 on immunoglobulins Ig-A (**A**) and Ig-G (**B**) in serum. The data were expressed as means ±SD (n = 10). The significant differences (*p* < 0.05) were marked as various superscript small letters (a–d).

As shown in Figure 4, the level of Ig-A and Ig-G of MC group significantly decreased (*p* < 0.05) compared with NC group. The serum Ig-A and Ig-G contents were significantly (*p* < 0.05) increased after the administration with CMP-1 and CMP-2. Meanwhile, a dose-dependent secretion of Ig-G and Ig-A was observed in CMP-1 and CMP-2 groups, respectively.

### 2.5. Effects of CMPs on Relative Gene Expression in Spleen

**Figure 5:**
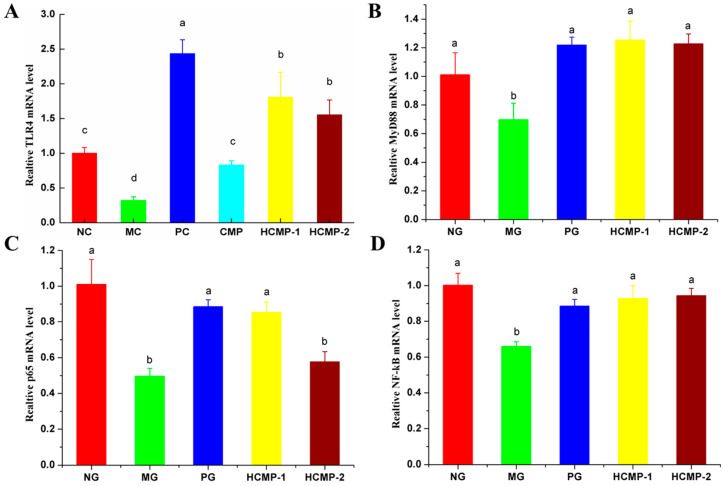
Effects of HCMP-1 and HCMP-2 on the mRNA expression of TLR4 (**A**), MyD88 (**B**), p65 (**C**) and NF-κB (**D**) in spleen. The significant differences (*p* < 0.05) were marked as various superscript small letters (a–d).

The mRNA expressions of TLR4, MyD88, p65 and NF-κB in the spleen were shown in Figure 5. High dose CMP-1 treatment could significantly induce the up-regulation of the mRNA expression of TLR4, MyD88, p65 and NF-κB, and the high dose CMP-2 treatment could significantly (*p* < 0.05) up-regulated the mRNA expression of TLR4, MyD88 and NF-κB.

### 2.6. Effects of CMPs on SCFAs Production

**Figure 6:**
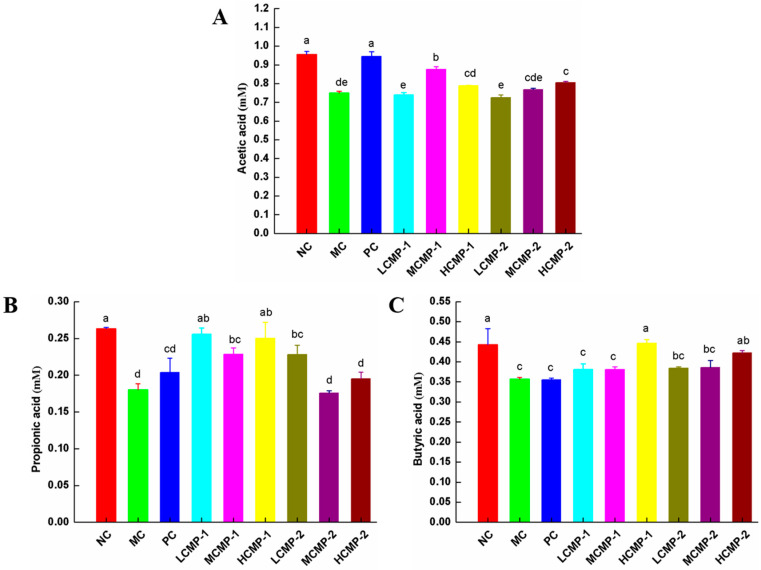
Effects of CMP-1 and CMP-2 on the contents of -acetic acid (**A**), propionic acid (**B**) and butyric acid (**C**) in the cecal contents. The significant differences (*p* < 0.05) are marked as various superscript small letters (a–e).

The contents of SCFAs in the cecum content were showed in Figure 6. It was obvious that acetic, propionic and *n*-butyric acids were the main SCFAs in the cecal content. Compared with the NC group, the contents of acetic, propionic and *n*-butyric acids of MC group were significantly decreased (*p* < 0.05). The levels of acetic in the MCMP-1 and HCMP-2 groups (Figure 6A), the levels of propionic in the three kinds of doses of CMP-1 and LCMP-2 groups (Figure 6B) and the levels of *n*-butyric acids in the HCMP-1 and three kinds of doses CMP-2 groups increased when they were compared with MC group (Figure 6C).

### 2.7. Effects of CMPs on the Gut Microbiota

**Figure 7:**
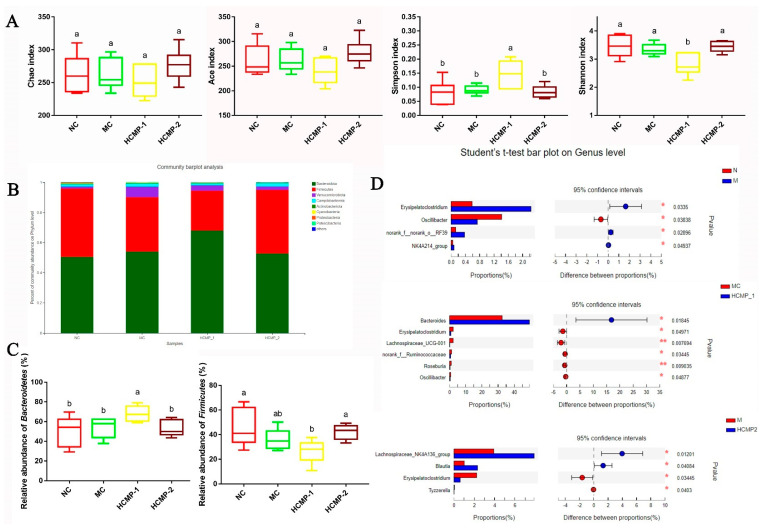
Effects of CMP-1 and CMP-2 administration on gut microbiota. (**A**) Alpha diversity evaluated from Chao, ACE, Shannon and Simpson indexes; (**B**) bacterial taxonomic profiling at the phylum level of the gut microbiota; (**C**) the relative abundances of *Bacteroidetes* and *Firmicutes.* One-way ANOVA procedure followed by the Dancan test was used to evaluate the statistical significance. The significant differences (*p* < 0.05) are marked as various superscript small letters. (**D**) Differentially the abundant of gut microbiota between the NC and MC, MC and HCMP-1, MC and HCMP-2 groups Wilcoxon rank sum test.

The effects of CMP-1 and CMP-2 on the alpha-diversity of gut microbiota were evaluated with Chao, Ace, Shannon and Simpson indexes by bacterial 16S rDNA pyrosequencing. The stable Rarefaction curves and Shannon curves showed that most of the bacterial diversity and new phylotypes were covered (data not shown). The four groups had a similar Chao and ACE indexes, which indicated that no significant differences in the richness of intestinal microorganism of them was found (Figure 7A). However, the high Simpson index and low Shannon index showed the CMP-1 group had the lowest diversity of intestinal microbes among the four groups.

As shown in Figure 7B, the gut microbiota of the four groups mainly consisted of *Bacteroidetes* and *Firmicutes* at the phylum level, accounting for almost 95% of the total bacteria. However, there was no significant difference among NC, MC and CMP-2 groups at the phylum level. The CMP-1 group showed the highest relative abundance of *Bacteroidetes* and the lowest relative abundance of *Firmicutes* (Figure 7C).

The microbiota compositions at genus level of the four groups were shown in Figure 7D. Compared with the NC group, the relative abundance of *Erysipelatoclostridum* significantly increased (*p* < 0.05), while the relative abundance of *Oscillibacter* significantly decreased (*p* < 0.05) in the MC group. Compared with the MC group, the decrease (*p* < 0.05) of *Erysipelatoclostridum* was detected in both CMP-1 and CMP-2 groups. In addition, the CMP-1 group showed higher level of *Bacteroides*, whereas *Lachnospiraceae*, *Ruminococcaceae*, *Roseburia*, *Oscillibacter* showed lower levels than those of the MC group. In addition, the CMP-2 group exhibited higher relative abundance of *Lachnospiraceae*, *Blautia* and lower relative abundance of *Tyzzerella*.

## 3. Discussion

The spleen and thymus were two vital immune organs. The spleen was a place for T and B cells colonization, immune response and synthesis of bioactive substances [21]. The thymus can regulate peripheral immune organs and immune cells, and provide differentiation and maturation of T cell [22]. Therefore, the spleen and thymus index reflects the level of immune function [10,23]. Generally speaking, the improvement of immunity is accompanied with immune organs index increases. In this study, the thymus index of MC group significantly decreased (*p* < 0.05), but the spleen index significantly increased (*p* < 0.05) compared with those of the NC group. Therefore, the pathological state of spleen tissue was further observed by HE staining. It found that the spleen tissue structure in MC group was changed as the boundary between red pulp and white pulp was blurred and the intercellular space became larger. The above results proved that the increase of spleen index was due to immune cell group damaged by CTX rather than the improvement of spleen immune function. The administration of CMP-1 and CMP-2 had no effect on thymus index but significantly decreased the spleen index and improved the internal structure of spleen, indicating that CMP-1 and CMP-2 could promote the development of spleen and recover the CTX immune damage.

Cytokines directly participate immune reactions by stimulating small soluble proteins which are synthesized by immune cells and non-immune cells [24]. Among them, Th1 and Th2 can secrete different types of cytokines to regulate the cellular and humoral immunity. The secretion of IL-2 and IL-6 can induce Th1 and Th2 cell responses [25]. IFN-γ is mainly produced by natural killer cells (NK) and Th1, and it is considered to be a part of innate and antigenic immunity. In addition, macrophages can secrete cytokines such as IL-6 and TNF-α to kill tumor cells by binding to polysaccharides or glycoproteins through a variety of receptors [26]. In this study, CMP-1 and CMP-2 could recover the original level of TNF-α, IL-2, IL-6 and IFN-γ in serum, which were significantly reduced by CTX. The results suggested that the administration of CMP-1 and CMP-2 could restore the immune function of immunosuppression mice induced by CTX through promoting the production of Th1 and Th2 cytokines.

Immunoglobulin is a kind of protein used to recognize and remove foreign bodies such as bacteria and viruses, which is mainly secreted by plasma cells [27]. When the immunosuppressive mice were treated with polysaccharide, Ig-A could produce to prevent the invasion of pathogens in the mucosal tissue [28]. Ig-G is the lowest molecular weight immunoglobulin, which can promote the phagocytic activity of macrophages and enhance immunity. The content of it in the serum can reflect the immune status of the body to pathogens [29]. Moreover, the serum immune globulins are essential markers of humoral immunity [30]. In our study, the serum levels of Ig-A and Ig-G in the MC group decreased significantly, indicating that the immune function of mice was damaged after CTX injection. While the administration of CMP-1 and CMP-2 increased the levels of Ig-A and Ig-G in serum, indicating that CMP-1 and CMP-2 enhanced humoral immune response of immunocompromised mice.

The spleen is an important organ for immune homeostasis, both innate and adaptive immune responses can be efficiently mounted in the spleen [31]. It is divided into white pulp and red pulp. The white pulp is rich in T lymphocytes, clonal expansion of activated B cells can take place in the B-cell zone in the white pulp [32]. Moreover, abundant B cells and plasma cells make the spleen the largest antibody producing organ, so as to regulate the serum antibody level [33,34]. In our study, CTX damaged spleen tissue, reduced the proportion of white pulp and blurred the boundary of tissue structure. It suggested that CTX has damaged T cells and B cells, which may lead to the decrease of immune effector factor content and antibody level in serum. In fact, it was true that CTX reduced the levels of cytokines (IL-2, TNF-α, IL-2, IL-6 and IFN-γ) and immunoglobulins in serum (Ig-G and Ig-A). However, the administration of CMP-1 and CMP-2 increased the contents of the above cytokines and immunoglobulins. According to the results of HE staining, to some extent, the spleen tissue was also repaired and the proportion of white pulp gradually increased. Which showed that the restored spleen provides a suitable place for the clonal proliferation of T and B cells, so that the anti-body level in serum also returns to the normal level. However, it is not clear what types of T and B cells affected by CMP-1 and CMP-2 to play an immunomodulatory role, and in-depth studies are needed to clarify how they work.

Polysaccharides can activate signaling cascades through pattern recognition receptors (PRRs) on the surface of immune cells [35], and it was detected in intestinal epithelial cells and macrophages of spleen, lymph nodes and bone marrow after oral administration [36]. Toll like receptor 4 (TLR4) is one of the important immune receptors on the surface of immune cells, which plays an important role in enhancing innate immune response and promoting the production of cytokines induced by polysaccharides [37]. TLR4 signaling pathway is a classic regulatory pathway, polysaccharides promote the production of cytokines, regulate immune response and anti-tumor by activates the cascade of immunoregulation pathway and key downstream transcription factors, such as MyD88 and NF-κBs [38,39,40]. In present work, the mRNA expression of TLR4, MyD88, p65 and NF-κB in the spleen of CTX-induced mice were significantly up-regulated by CMP-1, and the mRNA expression of TLR4, MyD88 and NF-κB also significantly up-regulated by CMP-2. It showed that CMP-1 and CMP-2 could enhance the immunity of immunocompromised mice through the TLR4/NF-κB signaling pathway in spleen. Guo et al. [39] also found that the polysaccharide from *Craterellus cornucopioides* (L.) Pers. exerted immunomodulatory effects by stimulating the expression of related proteins via TLR4/NF-κB signaling pathway.

Recent studies have found that polysaccharides can directly or indirectly act on the lower end of the intestine, and then regulate systemic immune response by changing the composition and abundance of intestinal microorganisms and promoting the production of short chain fatty acids. It was well known that most polysaccharides cannot be digested and absorbed in the stomach and small intestine, but they can be partially or completely hydrolyzed and metabolized into short chain fatty acids (SCFAs) by gut microbiota [41]. The important role of SCFAs is to provide energy for intestinal cells, promote the growth of intestinal cells, and regulate the innate immune system and adaptive immune system [40,42]. In this study, it was found that the content of acetic, propionic and *n*-butyric acids was increased after seven days of the administration of CMP-1 and CMP-2. The results of intestinal microbiological analysis with high dose CMP-1 could improve the relative abundance of beneficial bacteria *Bacteroidetes* and decrease the relative abundance of harmful bacteria *Firmicutes*. In addition, CMP-1 and CMP-2 could change the species richness abundance of intestinal microorganisms at the genus level. The research results showed that CMP-1 and CMP-2 might regulate immune response also depending on the modulation of the intestinal microbiota.

Although we have studied the relationship between the structure and activity of polysaccharides in previous studies [20], and preliminarily explored the immune activity mechanism of polysaccharides in this paper, the sugar moieties activity of polysaccharides has not yet been identified, and the research on the activity mechanism of polysaccharides is still insufficient. Because of the complexity of polysaccharide structure, a large number of structure-activity relationship studies and even the mechanism of action are only carried out in vitro [18,43,44]. Generally speaking, polysaccharides cannot be degraded and absorbed by the human digestive tract, and the conformation (advanced structure) of polysaccharides is affected by temperature, pH, solvent and so on, which is very important for their activity [45]. Therefore, further study needs to be conducted on how the characteristic of polysaccharides change in the body’s internal environment from mouth to intestine, basic active sugar moieties and mechanisms of polysaccharide in vivo. Moreover, whether or not those immunosuppressed individuals treated with the immunomodulators would be able to get a better immune response against a challenge from an antigen should also be studied.

## 4. Materials and Methods

### 4.1. Materials and Reagents

Dry *Poria cocos* was purchased from the local market of Luo tian, Hubei Province, China. CTX and Lentinan (the sugar content was 50%) were purchased from Shanghai Yuanye Biotechnology Co. Ltd. Shanghai, China. An amount of 4% paraformaldehyde solution was purchased from Wuhan Seville Biotechnology Co., Ltd. Wuhan, China. The Strand cDNA Synthesis Kit and AceQ qPCR SYBR Green Master mix (Q111-02/03) were purchased from Nanjing novozan Biotechnology Co., Ltd. Nanjing, China; acetic, propionic, *n*-butyric, *i*-butyric, *n*-valeric, *i*-valeric and 2-ethylbutyric acids were purchased from Aladdin Chemical Reagent Co., Ltd. (Shanghai, China). 

### 4.2. Preparation of CMP-1 and CMP-2

The preparation of CMP-1 and CMP-2 was performed according to Liu et al. [20]. Briefly, the mixture of *Poria cocos* powder and 85% ethanol was treated twice with NaOH for 0.5 h at 50 °C. Subsequently, the chloroacetic acid was added for substitution reaction for 6 h at 60 °C. Then, the glacial acetic acid was used to terminate the reaction. The precipitate was re-dissolved in distilled water, precipitated with 80% of ethanol and lyophilized to obtain crude carboxymethyl pachymaran (CMP). The crude CMP were further purified by DEAE-52 anion-exchange chromatography to obtain CMP-1 (85.60% of sugar content and 12.24% of uronic acids) and CMP-2 (84.52% of sugar content and 14.43% of uronic acids).

### 4.3. Animals and Experimental Design

Ninety female Specific PathogenFree (SPF) KM mice (20.0 ± 2.0 g, 4 weeks old) were purchased from the Laboratory Animal Centre of Huazhong Agricultural University (Wuhan, China, Certificate number: SCXK(Ei)2015-0019). All mice were housed in a controlled environment (temperature: 24 to 25 °C, humidity: 70–75%, and a lighting regimen of 12 h light-dark cycles) with free access to water. The animal experiment procedures were approved by the Committee of Animal Experimental Ethical Inspection of Laboratory Animal Centre, Huazhong Agriculture University (approved numble: HZUMO-2020-0052).

After one week accommodation, the animals were randomly divided into nine groups (n = 10) as follows: normal control (NC) group, model control (MC) group, positive control (Lentinan, PC) group, low-dose CMP-1 (LCMP-1), middle-dose CMP-1 (MCMP-1), high-dose CMP-1 (HCMP-1), low-dose CMP-2 (LCMP-2), middle-dose CMP-2 (MCMP-2) and high-dose CMP-2 (HCMP-2) group. Except NC group, other eight group were intraperitoneally injected with 80 mg/kg/d of CTX for three days to induce immunosuppression, and the NC was intraperitoneally injected with equivalent normal saline. After that, the mice of PC, LCMP-1, MCMP1, HCMP-1, LCMP2, MCMP-2 and HCMP-2 were administered with 200 mg/kg/d Lentinan, 50, 100, 200 mg/kg/d CMP-1 and 50, 100, 200 mg/kg/d CMP-2 for next one week, respectively, while the NC and MC were administered with equivalent normal saline. During the experiments, the body weight of mice were measured every day. After the last administration, the mice were fasting for 12 h but drank water freely. The mice were sacrificed by cervical dislocation after collecting blood samples, the collected tissues and serum were stored in −80 °C for further analysis.

### 4.4. Determination of Organ Index

The mice were weighed before sacrifice. The thymus and spleen were surgically excised, connective tissue and fat on the surface were removed and weighed. The immune organ index was calculated with the following formula:

(1) $$Spleen\;or\;thymus\;Index=\frac{spleen\;or\;thymus\;weight\left(mg\right)}{body\;weight\left(g\right)}$$

### 4.5. Pathological Examination of Immune Ogran

The spleen samples were fixed in 4% paraformaldehyde. The sample were embedded, sectioned and stained with hematoxylin-and-eosin-stained histopathological (HE), and then photographed with an inverted microscope for histological evaluation.

### 4.6. Measurement of Serum Immunoglobulin and Cytokines

The blood samples of each group were collected in heparinized tubes by removing the eyeballs before the mice were sacrificed by cervical dislocation. Then the blood samples were centrifuged at 4 °C, 3000 rpm for 10 min to separate the serum. The serum samples were divided into single dose as needed at 4 °C and then stored at −80 °C for further analysis. The level of cytokines (IL-2, IL-6, TNF-α and INF-γ) and immunoglobulin (Ig-A and Ig-G) were determined using ELISA kits (Shanghai Yuchun Biotechnology Co., Ltd., Shanghai, China) according to the manual.

### 4.7. RNA Extraction and Real-Time Quantitative PCR

Total RNA was extracted from spleen using TRIzon (Beijing Kangwei century Biotechnology Co., Ltd., Beijing, China). The purity (A_260_/A_280_) of the RNA and its concentration were evaluated by a Nanodrop 2000 (Thermo Scientific, Waltham, MA, USA). Then, the total RNA was used for reverse transcription according to the Strand cDNA Synthesis Kit (Vazyme Biotech Co., Ltd., Nanjing, China) to obtain cDNA, and the cDNA samples were incubated with AceQ qPCR SYBR Green Master mix (Vazyme Biotech Co., Ltd., Nanjing, China) with the following thermal cycling conditions: 95 °C for 5 min, followed by 40 cycles of 95 °C for 10 s, 60 °C for 30 s. The relative quantitative expression of genes of TLR4, MyD88, p65 and NF-κB mRNA were analyzed by 2^−ΔΔCt^ method on the Quantstudiotm 6 flex (ABI, New York, NY, USA) using β-actin as an internal reference [46].

### 4.8. Determination of Contents of Short Chain Fatty Acids

Used the method in Cheng, Wu, Chu, Tang, and Xu [47] with minor modifications. The contents of short chain fatty acids (SCFAs) including acetic, propionic, *n*-butyric were determined using an Agilent 6890 N gas chromatography (GC) system equipped with a DB-FFAP column (30 m × 0.25 mm × 0.50 μm, Agilent, Santa Clara, CA, USA) and a flame ionization detector (FID, Agilent, Santa Clara, CA, USA). Briefly, the cecal content (100 mg) was mixed with 1 mL of deionized water and shaken at 4 °C for 1 h. The mixture was centrifuged at 4 °C, 12,000 rpm for 15 min. and 0.8 mL of supernatant was mixed with 50% H_2_SO_4_ and 8 µL 2-ethylbutyric acid (internal standard). The mixture was acidified at 4 °C for 10 min and then extracted by 0.8 mL of ether at 4 °C for 30 min. The mixture was centrifuged at 4 °C, 12,000 rpm for 5 min, and the supernatant was filtered through a 0.45 μm membrane for SCFAs analysis. The operating conditions were set as follows: the initial column temperature was kept at 105 °C for 3 min, then increased to 170 °C at a rate of 10 °C/min and kept at 170 °C for 2 min, next increased to 230 °C at a rate of 20 °C/min and kept at 230 °C for 2 min, the split ratio was 10:1 and the flow rates of N_2_, H_2_ and air makeup gas were set at 25, 40 and 400 mL/min, respectively.

### 4.9. Gut Microbiota Analysis

The effect of CMP-1 and CMP-2 on the gut microbiota was analyzed with 16S rRNA Illumina sequencing technology. The genomic DNA of fecal sample of NC, MC, HCMP-1 and HCMP-2 was extracted. The quality, concentration and integrity of DNA were detected by NanoDrop2000 and agarose gel. Then, the amplification of the V3-V4 region of the bacterial 16S rRNA gene was performed with the Primer 338F: Illumina adapter sequence 5′-ACTCCTACGGGAGGCAGCAG-3′ and Primer 806R: Illumina adapter sequence 5′-GGACTACHVGCCTWTCTAAT-3′. The sequencing and bioinformatics study of fecal samples were performed by Shanghai Majorbio Bio-pharm Technology Co., Ltd. (Shanghai, China) on the Illumina MiSeq platform to generate 2 × 250 bp paired-end reads.

### 4.10. Statistical Analysis

All of the experimental data are presented as mean ± standard deviation (SD). The statistical significance of difference with ANOVA was analyzed by SPSS 18.0. (IBM, Armonk, NY, USA) Duncan’s test was used for the assay of differences (*p* < 0.05). The resulting sequences were clustered to operational taxonomic units (OTUs) with 3% divergence (97% similarity) using Parse (v.7.1, Majorbio, Shanghai, China) for further bioinformatics analysis. The data of gut microbiota were performed with the Majorbio Cloud Platform (http://www.majorbio.com, accessed on 10 August 2021).

## 5. Conclusions

In summary, the present study demonstrated that the CMP-1 and CMP-2 could promote the development of spleen (alleviating immune organ damage and reducing spleen indexes) and restore the content of cytokines TNF-α, IL-2, IL-6 and IFN-γ and immunoglobulin Ig-A and Ig-G in immunocompromised mice induced by CTX. In addition, CMP-1 and CMP-2 could regulate the mRNA expression of TLR4, MyD88, p65 and NF-κB in the spleen. Furthermore, the administration of CMP-1 and CMP-2 enhanced the production of SCFAs and modulated the relative abundance of the gut microbiota at the genus level. Our results indicated that CMP-1 and CMP-2 exhibited immune-protective effects on CTX-treated mice. Moreover, CMP-1 and CMP-2 might play an immunomodulatory activity by regulating the gene expression in the TLR4/NF-κB signaling pathway and modulating the composition and abundance of intestinal microbiota.

## Data Availability

Not applicable.

## Sample Availability

Samples of the compounds carboxymethyl pachymaran are available from the authors.
